# A river ran through it: Floodplains as America’s newest relict landform

**DOI:** 10.1126/sciadv.abo1082

**Published:** 2022-06-24

**Authors:** Richard L. Knox, Ryan R. Morrison, Ellen E. Wohl

**Affiliations:** 1 Department of Geosciences, Colorado State University, Fort Collins, CO, USA.; 2 Department of Civil and Environmental Engineering, Colorado State University, Fort Collins, CO, USA.

## Abstract

Artificial levees are a major human modification of river corridors, but we still do not have a clear understanding of how artificial levees affect floodplain extent at regional and larger scales. We estimated changes in river-floodplain connectivity due to artificial levees in the contiguous United States (CONUS) using a combination of artificial levee databases, delineations of floodplain areas, and deletion of artificial levees from topography. Our results indicate that artificial levees do not only decrease floodplain extent but also alter locations of floodplain connectivity. Anthropogenically connected and disconnected locations are similar in land cover and are predominantly, in decreasing order of extent, cultivated, wetland, forested, and developed land cover types, with more than 30% of the entire floodplain area in the CONUS cultivated or developed. This study indicates that artificial levees cause complex changes in river-floodplain connectivity and can increase flooded areas in some rivers.

## Teaser

This analysis of artificial levee impacts on floodplains in the contiguous United States reveals some unexpected results.

## INTRODUCTION

River corridors include the active channel(s), floodplain, and underlying hyporheic zone. We define a floodplain as a frequently flooded, low-relief landform created by erosional and depositional processes under the contemporary hydrologic regime (*1*). River scientists and engineers emphasize the importance of three-dimensional connectivity within river corridors (*2*). Examination of lateral connectivity between the channel and floodplain can focus on water in association with flooding hazards (*3*), flood peak attenuation (*4*), floodplain inundation [e.g., perirheic zones (*5*)], ecological considerations [e.g., flood pulse concept (*6*)], sediment fluxes (*7*–*10*), or other processes, but the commonality is that alteration of natural levels of lateral connectivity influences diverse river corridor functions.

American floodplain development kept pace with flood protection efforts during the 20th century, resulting in the constant rise of average flood-related economic losses (*11*). Worldwide, the restoration, rehabilitation, and conservation of large floodplain rivers are increasingly in conflict with development (*12*, *13*). Managing these conflicts requires an understanding of floodplain location and extent, as well as the water and sediment interactions between floodplain and channel (*13*, *14*). A rapid increase in the availability of Earth observation datasets and computational power has created new opportunities for the evaluation of floodplain mapping models (*15*), including hydrodynamic models at the continental scale (*16*) and hydrogeomorphic models at basin, continental, and global scales (*14*, *15*, *17*–*19*). Hydrodynamic models are the state-of-the-art method for flood hazard analysis and include backwater effects, flood wave attenuation, and urban interactions (*15*). Hydrogeomorphic models make efficient use of topographic data and are based on the natural depiction of floodplain topography resulting from recurring floods (*20*). The level of agreement between hydrogeomorphic models and other flood hazard models indicates the suitability of hydrogeomorphic modeling, especially in data-poor areas (*21*). However, one of the sources driving model disagreement and inaccuracy is infrastructure, including artificial levees (*14*, *19*).

Diverse human activities alter flow regime, floodplain morphology, and channel-floodplain connectivity (*22*, *23*). Artificial levees, for example, are built to inhibit lateral connectivity and are associated with substantial ecological harm (*24*, *25*). Unexpectedly, there are few studies that evaluate the impact of artificial levees on floodplain extent at large watershed scales (*18*). One example of such an evaluation used the hydrogeomorphic GFPLAIN flood model (*17*) on two versions of a DEM (digital elevation model), one with artificial levees removed, in the 100,000-km^2^ four-digit hydrologic unit code (HUC) (table S1) Wabash basin (*18*). At the continental scale, however, it remains unknown to what extent floodplains have been disconnected from channels in the United States or elsewhere in the world.

This is in notable contrast to knowledge of longitudinal disconnectivity created by dams [e.g., (*26*–*28*)]. Dams are more readily detected in remote imagery, and there are more likely to be systematic records of dam construction and the dimensions of individual dams (e.g., the U.S. Army Corps of Engineers’ National Inventory of Dams or Global Dam Watch’s global dam database). Increasing recognition of the intensity and spatial extent of river longitudinal disconnection by dams has been accompanied by a growing scientific literature on the environmental hazards created by this disconnectivity [e.g., (*29*–*32*)]. Extensive networks of artificial levees may be creating a similar amount of riverine degradation, but remotely delineating natural floodplains remains difficult, especially on smaller rivers [e.g., (*33*, *34*)], and efforts to quantify the lateral disconnection of floodplains by artificial levees at regional to continental scales have been limited by lack of systematic records and inability to detect levees in remote imagery.

The U.S. Army Corps of Engineers and the Federal Emergency Management Agency (FEMA) maintain a national levee database (NLD) for the United States, but it has not been evaluated for completeness until recently. In an earlier paper, we estimated the completeness of the NLD to be 20.4%, with more than 182,000 km of undocumented potential levees identified in the contiguous United States (CONUS) (*35*).

Here, we explore the spatial extent of lateral disconnectivity caused by artificial levees, called “anthropogenically disconnected” floodplains, as well as areas that levees cause to flood, called “anthropogenically connected” floodplains, in the CONUS. Anthropogenically disconnected floodplains are former floodplain areas that are no longer connected to stream flow because of artificial levee construction. Anthropogenically connected floodplains are areas that were not connected or less frequently connected to stream flow formerly but now are more likely to inundate because of artificial levee construction. We apply a GFPLAIN flood model calibrated with FEMA flood hazard maps (table S1) to two DEMs: one unmodified and one with artificial levees removed. Our primary objectives are to determine the spatial distribution and stream order patterns of floodplain disconnection by artificial levees in the CONUS.

## RESULTS

### Area analysis of anthropogenically connected and disconnected floodplain areas

**Fig. 1.**
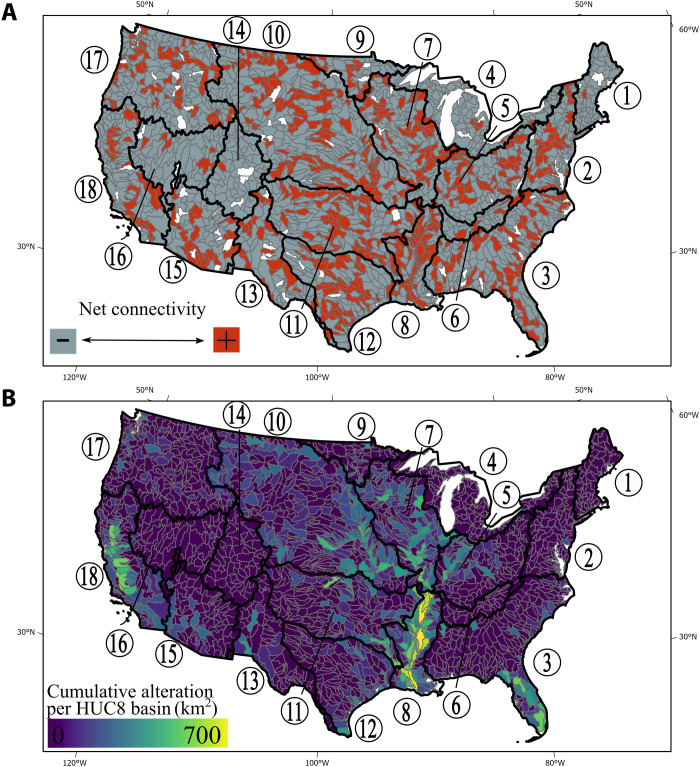
Net connectivity and cumulative alteration in the CONUS HUC8 basins. (**A**) Net connectivity compares whether each HUC8 basin has more anthropogenically connected or more anthropogenically disconnected floodplain area. Basins with no change in connectivity are indicated by white. (**B**) Cumulative alteration by anthropogenically connected and disconnected floodplain areas. The 18 HUC2 basins are annotated in each figure with black lines and by numbers.

The net effect of artificial levees varies by HUC8 basin with anthropogenically connected (areas flooded by artificial levees) exceeding anthropogenically disconnected floodplains (floodplains separated from rivers by artificial levees) CONUS-wide (Fig. 1A and table S2). At the larger HUC2 basin scale, the Lower Mississippi River (LMR) (HUC2 no. 8, 6714 km^2^), California (HUC2 no. 18, 2043 km^2^), and Missouri basins (HUC2 no. 10, 2016 km^2^) had the greatest total anthropogenically connected and disconnected floodplains (Fig. 1B). These basins have the greatest (46,569 km), fourth greatest (23,222 km), and second greatest (43,659 km) lengths, respectively, of known and potential artificial levees (*35*).

### Land cover analysis

**Fig. 2.**
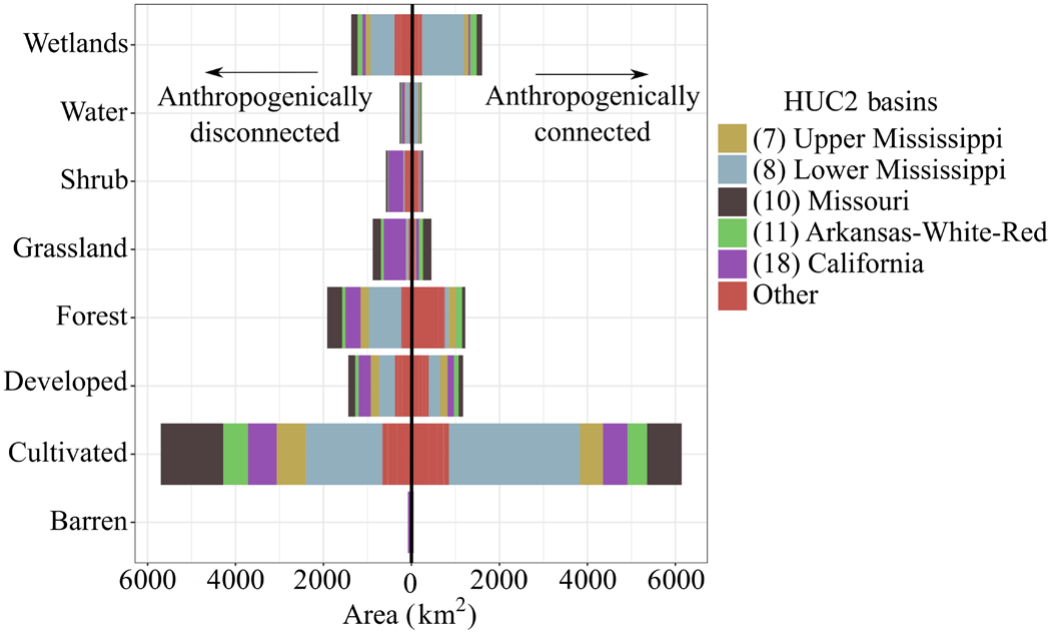
Land cover of anthropogenically disconnected floodplain and anthropogenically connected areas. CONUS land cover area (square kilometers) of anthropogenically disconnected floodplain and anthropogenically connected areas with HUC2 basin contributions annotated by color. HUC2 basins contributing less than 1000 km^2^ of cumulative alteration were combined as “Other” for clarity.

Land cover patterns of anthropogenically connected and disconnected floodplains are similar but with some notable differences (Fig. 2 and table S4). By far, cultivated land cover (cultivated crops and hay/pasture) makes up the largest proportion (55% for anthropogenically connected and 47% for anthropogenically disconnected floodplain) of each type of area. Wetlands (15% anthropogenically connected and 11% anthropogenically disconnected floodplain), forested (11 and 16%), and developed (11 and 12%) categories constitute progressively smaller proportions of land cover.

There are several notable differences in the anthropogenically connected and disconnected floodplains (referred to as “disagreement areas”) when compared to the agreement areas (table S4). Cultivated land cover constitute twice the size of disagreement areas (55 to 47%) when compared to agreement areas (24%). Forested and developed areas experience similar trends. Agreement areas include more wetlands, open water, and shrub cover.

### Stream order analysis

**Fig. 3.**
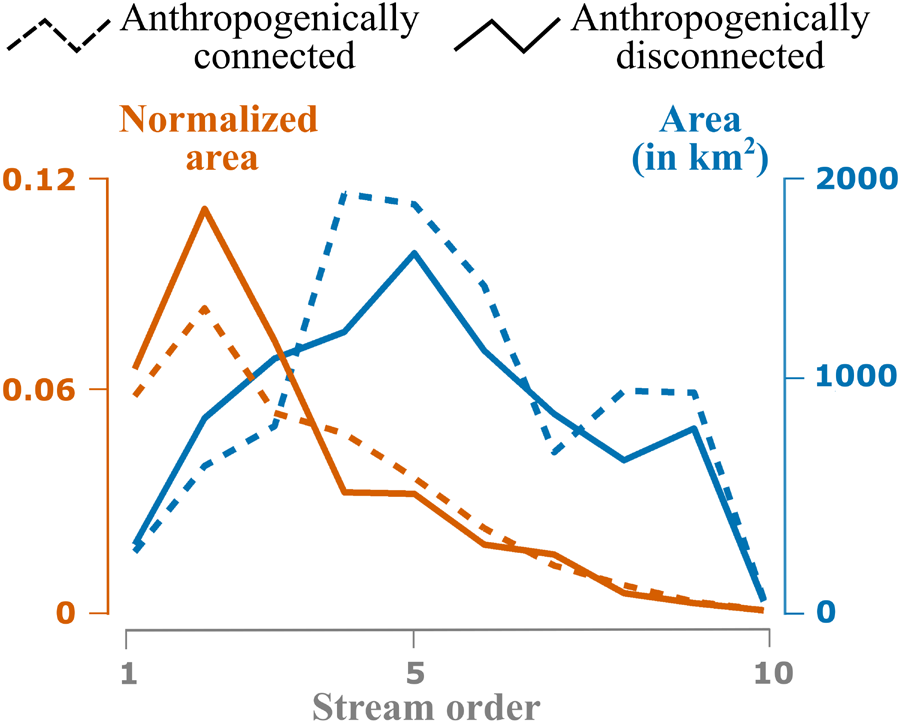
Stream order analysis of anthropogenically connected and disconnected areas. Actual and normalized areas in the CONUS, distinguished by stream order. Areas are normalized by stream order contributions to the agreement areas.

Stream order is a metric used to classify streams: A first-order stream has no tributaries, and stream order increases downstream from the confluence of two streams of equal order (*36*). Artificial levees are more likely to disconnect floodplains in first- to third-order streams, whereas the levees are more likely to enhance floodplain inundation in streams of fourth and higher orders (Fig. 3). Stream order contribution patterns vary widely by HUC2 basin (fig. S1). When compared to stream order contributions to agreement areas, disagreement areas peak in order two streams and then decrease with increasing stream order, indicating the effects of artificial levees on smaller order streams (Fig. 3).

## DISCUSSION

Our finding that the anthropogenically connected extent was larger than the anthropogenically disconnected floodplain extent (table S2) was unexpected, although the 811-km^2^ difference was much less than 1% of the agreement area floodplain. This corroborates other research illustrating the unintended upstream and downstream flooding caused by artificial levees [e.g., (*37*–*40*)].

**Fig. 4.**
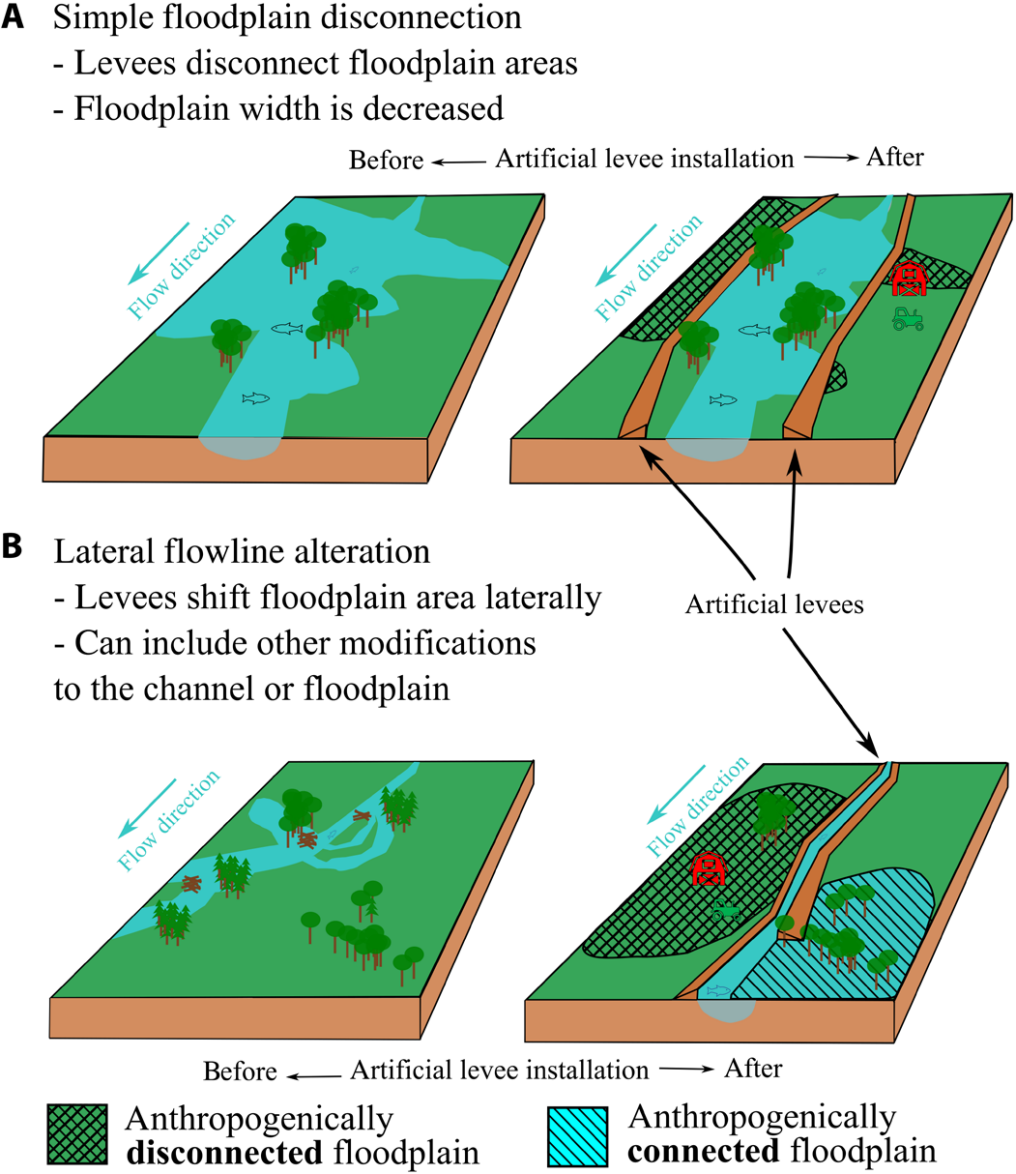
Two examples of floodplain alteration before and after artificial levee installation. (**A**) In simple floodplain disconnection, levees disconnect floodplains and rivers. (**B**) Lateral flowline alteration occurs when levees alter the spatial extent of floodwaters, causing areas to flood (called anthropogenically connected floodplain) and areas to disconnect (called anthropogenically disconnected floodplain). This type of alteration can occur with other modifications to include channelization, rerouting of tributary inputs, levee construction on one side of the stream only, and cut and fill from channel or levee construction.

Where artificial levees disconnect floodplains, their presence affects active floodplain area through two processes: simple floodplain disconnection and lateral flowline alteration (Fig. 4). The former occurs when artificial levees disconnect floodplains and river channels, especially along larger stream systems (Fig. 4A). The end result of this process is a reduction in active floodplain area. The latter, lateral flowline alteration, involves an adjustment of the direction of flood waters and shifts the location of flooding (Fig. 4B). Instead of decreasing the active floodplain, floodplain location is shifted from one location to another. In this example, the course of the river channel is adjusted and channelized through an artificial channel with levees. The result is the disconnection of the former channel and floodplain from floodwaters. Floodwaters are conveyed to the bottom of the figure where the channel is leveed on one side only, resulting in both anthropogenically connected and disconnected floodplains. With the exception of one other study (*18*), this effect of artificial levees on floodplain extent has gone unreported until now, despite the well-known ability of levees to increase stage height (*39*). This type of alteration is a result of the massive degree of topographic adjustment represented by the construction of enough artificial levees to wrap around Earth six times (*35*). The concentration of artificial levees along smaller streams (73% of artificial levees are along streams of orders 2 to 6) (*35*) indicates the ability of this process to affect floodplain connectivity in ways that do not fit the normal conceptual model of artificial levees, which is based on larger stream systems [e.g., (*39*)]. The discovery of lateral flowline alteration in addition to the traditional understanding of simple floodplain disconnection is the latest facet of our understanding of the Anthropocene.

Gilbert White noted that the main policy aim of the last century was to minimize losses on floodplains instead of maximizing social benefits (*11*). Despite that aim and the expenditure of billions of dollars on flood protection projects, flood losses in the United States continued to rise and were 2.5 times higher during the period 1951–1985 than 1916–1950 (*37*). What insight can this study provide to this problem? We found that if we considered cultivated (cultivated crops and hay pasture) and developed land covers as those susceptible to economic losses, then those areas cover 297,794 km^2^, which is 66, 59, and 30% of the anthropogenically connected floodplain, anthropogenically disconnected floodplain, and agreement area floodplains, respectively. These estimates corroborate recent research indicating the large-scale conversion of Mississippi River basin floodplains to cultivated and developed land covers during the past 60 years (*41*). The preeminence of cultivated land covers affected by artificial levees in the CONUS reflects the intersection of the huge concentration of levees in the Mississippi basins (40% of levee length in the CONUS is in the Lower and Upper Mississippi basins) (*35*), with the degree of agricultural intensification in the same basins (*25*). The association of wetland drainage with cultivation (*12*, *25*) indicates the reason for the disconnection of more than 1500 km^2^ of wetlands by artificial levees (Fig. 2). These trends also reflect artificial levee association with certain land covers, with 67% of levees situated on developed or cultivated land covers in the CONUS (*35*). Cultivated and developed land covers constitute 30.6% of floodplain areas and 3.7% of the entire CONUS. The fact that nearly one-third of floodplain areas in the CONUS are used for some sort of economic purpose likely explains at least one of the causes for the trend noted by Tobin and White (*11*, *37*).

The anthropogenically connected and disconnected floodplain areas in the Lower Mississippi basin are notable given their large magnitude and the size difference, with the anthropogenically connected areas ~70% larger than the anthropogenically disconnected floodplain areas (table S2). This estimate, that the area flooded by artificial levees is 70% larger in extent than areas “protected” by levees, deserves some exploration. Each of these areas is created by floodwaters with the same upstream contributing area. An analogy is pouring one cup of water into a shallow bowl and then again into a tall, narrow vase. The same amount of water results in a different cross-sectional area. Therefore, we tested the idea that floodplain geometry differences are responsible for the seemingly large difference in extent. We generated slope maps for the unmodified and modified DEMs and calculated the maximum and median values in each floodplain segment. The anthropogenically disconnected floodplain segments experienced greater slope, despite having artificial levees removed from their margins (table S3).

This supports the idea that, in the LMR basin, more confined anthropogenically disconnected floodplain areas result in a smaller floodplain extent given the same contributing area and reflects the different processes that formed each area. Even in a dynamic system such as the LMR, anthropogenically disconnected floodplains are formed by fluvial and floodplain processes operating over hundreds of years. Anthropogenically connected areas have only recently experienced the same processes. We contend that similar dynamics, with artificial levees altering flow paths across a heterogeneous topography, result in the differences apparent in table S2 between anthropogenically connected and disconnected floodplain areas.

The limitations of these results include the application of the hydrogeomorphic floodplain model in areas with characteristics that can lead to lower model accuracy [e.g., dry, steep, flat areas or those near the coast] (*19*, *21*). Calibration of the floodplain model at the two-digit HUC basin level provides some mitigation. Other limitations include the current inability to ground-truth potential levees from the study by Knox *et al.* (*35*) and the absence of a stream order–dependent buffer size for topography modification.

We removed known and potential artificial levee locations from a modified 1–arc sec DEM of the CONUS. We then generated two hydrogeomorphic floodplains using the modified and unmodified DEM and compared the location and area, land cover, and the stream order of rivers associated with each floodplain segment. The overall effect of artificial levee removal was not to just extend the floodplain but rather to shift the location of flooding. The massive extent and length of artificial levees, especially along smaller streams (*35*), require us to realize that floodplain alteration by artificial levees extends beyond normal conceptions of embankment. Constructed by individual farmers, municipal boards, and state and federal agencies over a 300-year period (*22*), artificial levees constitute a massive topographical alteration at the CONUS level that alters floodwater flow paths, especially along smaller streams. This previously unknown dimension of artificial levee impacts to floodplains illustrates that we have massively underestimated the ecological and hydrological damage of levees. Anthropogenically disconnected floodplain (protected from flooding) and anthropogenically connected (induced to flood by artificial levees) areas each accounted for about 1% of the total CONUS floodplain, which was more than 960,000 km^2^. More than 60% of the disagreement areas (mapped floodplain that differed with and without artificial levee presence) were cultivated, forested, wetland, or developed land cover. More than 30% of the CONUS floodplain was either cultivated or developed. These results corroborate, on a national scale, previous local-scale investigations of the unintended consequences of artificial levees.

## MATERIALS AND METHODS

### Experimental design

Our analyses included the following major steps. First, we generated GFPLAIN floodplain areas for each of the 18 two-digit HUC (HUC2) basins in the CONUS using the 30-m resolution USGS National Elevation Dataset (“Extended methods” section in the Supplementary Materials). Then, we altered the topography in each basin by deleting known and potential levees from the topography and applied GFPLAIN to the modified topography. Last, we analyzed the differences in floodplain extent for the unmodified topography and the modified topography by stream order, land cover, and area.

### Topography modification

This procedure is similar to DEM modification by Scheel *et al.* (*18*) in which the topographic effect of levees are removed from the DEM (fig. S2). We developed an ArcGIS Pro (*42*) model that separately modifies topography near NLD levees and near potential levees from Knox *et al.* (*35*) before combining results into one DEM. The same procedure is applied to both types of levees. First, the centerline of each levee is identified. Then, the centerline is buffered by 90 m. This area, within 90 m of the centerline, is the only area in which topography is adjusted during the process. The 90-m buffered area is deleted from a second larger buffered area of 150 m beyond the original 90-m buffer, creating a ring of unmodified topography varying in distance of 90 to 240 m from the levee centerline. The focal mean tool, with a radius of 120 m, is then applied to the area of the original 90-m centerline buffer using the values of the ring of unmodified topography. Last, three separate DEMs are combined together using the minimum value and the mosaic tool: the unmodified DEM, the modified DEM using NLD centerlines, and the modified DEM using potential levee centerlines.

### Statistical analysis

We developed custom ArcGIS Pro (*42*) and RStudio (*43*) scripts to analyze the differences between the GFPLAIN floodplain extent developed from unmodified and modified topography. Working by HUC2 basin, we identified areas of agreement and disagreement. Our analysis focused mainly on the latter because areas of disagreement are created solely by the presence or removal of artificial levees. Areas of disagreement between the two floodplains were classified as either anthropogenically disconnected floodplain or anthropogenically connected and were analyzed using ArcGIS Pro. Anthropogenically disconnected floodplains are those separated from overbank flow by the installation of artificial levees. Anthropogenically connected areas are those that are caused to flood by the installation of artificial levees. These areas were measured in terms of square kilometers, and their coverage in the 2016 National Land Cover Database (table S1) was determined in ArcGIS Pro. We determined the largest stream order associated with each floodplain segment by searching in ArcGIS Pro within 500 m of each segment for every stream segment in the National Hydrography Dataset (table S1). We selected the largest stream order per floodplain segment in RStudio using the map_dfr function in the purrr package (*44**)* as well as the group_by and summarise functions in the dplyr package (*45**)*. We chose 500 m as the search radius after using several smaller values in the LMR HUC2 basin and determining that this search radius connected NHD segments with most floodplain segments (*n* ~ 60,000 of 66,000 total segments) without connecting unrelated stream and floodplain segments.

## Supplementary Materials

### This PDF file includes:

Supplementary Text Figs. S1 to S3 Tables S1 to S4 References
