# Supplementary material for: A river ran through it: Floodplains as America’s newest relict landform
Source: Sci Adv. 2022 Jun 24;8(25):eabo1082. doi: 10.1126/sciadv.abo1082 (PMC9232114; doi:10.1126/sciadv.abo1082)
Supplement: Supplementary file 1 — Supplementary Text Figs. S1 to S3 Tables S1 to S4 References [file sciadv.abo1082_sm.pdf]

Science Advances
AAAS

Supplementary Materials for

A river ran through it: Floodplains as America's newest relict landform

Richard L. Knox *et al.*

Corresponding author: Richard L. Knox, richknox@colostate.edu

Sci. Adv. **8**, eabo1082 (2022)
DOI: 10.1126/sciadv.abo1082

This PDF file includes:

Supplementary Text
Figs. S1 to S3
Tables S1 to S4
References

Supplementary Text

Extended methods

Floodplain model calibration

We delineated floodplains using the hydrogeomorphic floodplain delineation tool, GFPLAIN (*20, 47*), which runs as three python scripts in ArcGIS Pro. There are two main steps (4): DEM pit filling, determination of flow direction and cell accumulation, and identification of the river network based on contributing area threshold, and (19) flow height estimation along the network based on the upstream contributing area and the following scaling relationship based on Leopold and Maddock (*48*):

$$FH = aA^b \tag{1}$$

where FH is the flow depth for the *A* contributing area, with *a* and *b* dimensionless scaling parameters (*18*). Regional flow depth – contributing area scaling laws are especially valid (*19*), so we calibrated the scaling parameters in each 2-digit HUC basin with FEMA flood map areas A and AE (*SI Appendix*, table S1) along streams of orders one through six, as described in more detail by Knox *et al.* (*35*). We selected 50 km^2 for the contributing area threshold, based on previous research (*15, 18*) and applied GFPLAIN separately in each HUC2 basin.

Stream order contribution normalization

We compared stream order contribution to disagreement areas to that in the agreement areas using the following equation, where "X" indicates the stream order and "disagreement area" is either anthropogenically connected [AC] or anthropogenically disconnected [AD] floodplain:

$$\begin{aligned} &Area\ contribution\ normalized\ by\ agreement\ area \\ &\quad = \frac{stream\ order\ X\ contribution\ to\ disagreement\ area}{stream\ order\ X\ contribution\ to\ agreement\ area} \end{aligned} \tag{2}$$

Normalized difference metric

We developed a metric, normalized difference, to indicate the type of alteration most prevalent in a HUC8 or HUC2 basin.

$$Normalized\ difference = \frac{(Sum\ of\ AD\ area) - (Sum\ of\ AC\ area)}{(Sum\ of\ AD\ area) + (Sum\ of\ AC\ area)} \tag{3}$$

This metric ranges from -1, indicating completely artificially connected, to +1, indicating completely anthropogenically disconnected floodplain. We calculated this metric for each HUC2 basin by determining the mean value of HUC8 basins there.

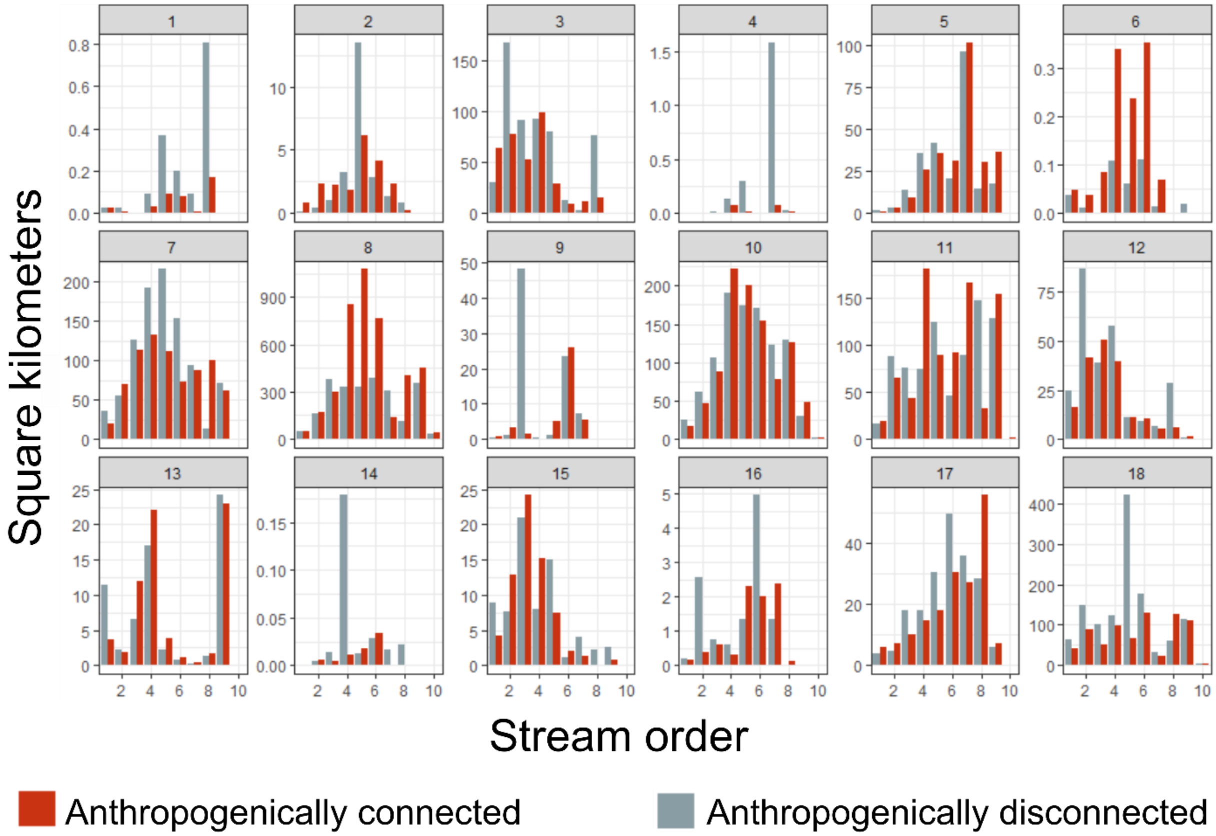

Fig. S1.

Area of stream order contribution to anthropogenically connected and disconnected floodplain areas by HUC2 basin.

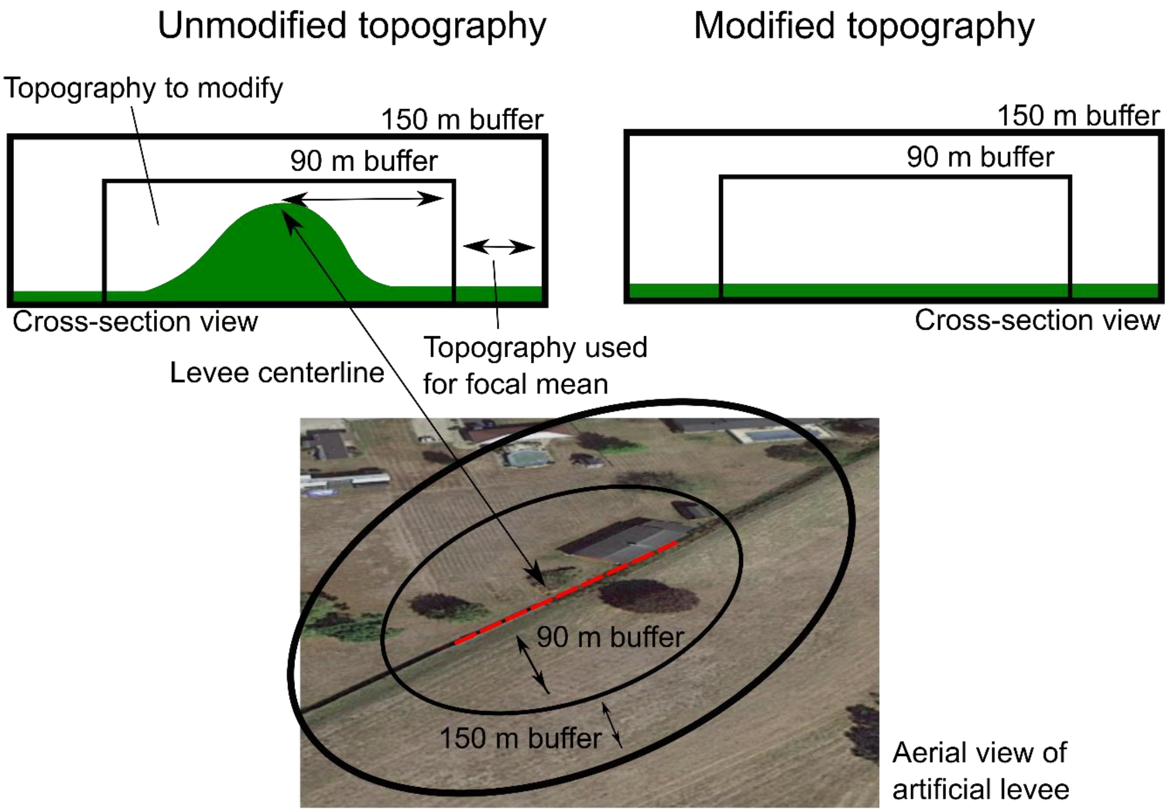

Fig. S2.

Topography modification for NLD and potential levees. The topography within 90 m of the levee centerline is modified by applying a focal mean with a 120 m radius using only the topography between the 90 m and 150 m buffers.

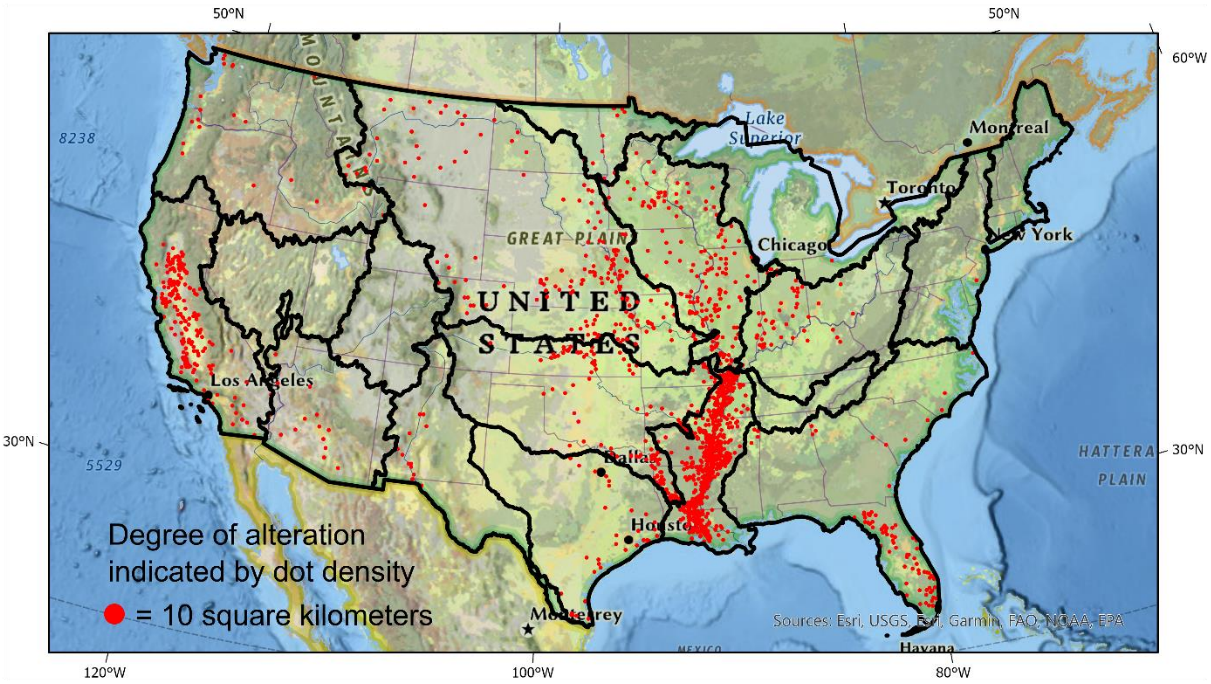

Fig. S3.

Cumulative alteration by anthropogenic floodplain disconnection and connection where the degree of alteration is indicated by dot density, with one dot representing 10 square kilometers.

Table S1.

Sources and descriptions of data used in study.

Data	Description	Source
Elevation Derivatives for National Applications (EDNA)- DEM	30-m resolution DEM	(*49*)
National hydrography dataset (NHD) High Resolution	National stream location and orders	(50)
FEMA Flood maps	"A" and "AE" flood zones indicating the 100 year recurrence interval	https://msc.fema.gov/portal/advanceSearch
1:250,000-scale Hydrologic Units of the United States	2-digit Hydrologic Unit Code (HUC2), Watershed Boundary Dataset units	(51)
National levee dataset (NLD)	known locations of artificial levees	https://levees.sec.usace.army.mil/
Potential levees	potential locations of artificial levees not in the NLD	https://doi.org/10.4211/hs.729c0aea00bb48d6b6814c147e4318c4
Land Cover (NLCD)	National Land Cover Database, 2016, 30-m resolution	(52)

Table S2.

Area in square kilometers of each type of area and the normalized difference by HUC2 basin.

**HUC2 basin**	**Agreement (km^2)**	**Anthropogenically connected (km^2)**	**Anthropogenically disconnected (km^2)**	**Normalized difference**
New England (1)	13,463	1	2	0.65
Mid-Atlantic (2)	33,647	20	23	0.23
South Atlantic-Gulf (3)	223,063	378	575	0.16
Great Lakes (4)	25,378	10	11	0.18
Ohio (5)	32,939	273	244	0.08
Tennessee (6)	8,850	1	0	0.30
Upper Mississippi (7)	73,917	786	971	0.06
Lower Mississippi (8)	117,658	4,252	2,462	0.00
Souris-Red-Rainy (9)	22,767	44	83	0.07
Missouri (10)	81,588	992	1,024	0.11
Arkansas-White-Red (11)	59,114	850	803	0.08
Texas-Gulf (12)	84,760	190	273	0.09
Rio Grande (13)	29,765	70	68	0.15
Upper Colorado (14)	12,168	0	0	0.36
Lower Colorado (15)	28,366	82	86	0.10
Great Basin (16)	37,210	9	12	0.33
Pacific Northwest (17)	40,731	179	197	0.12
California (18)	31,730	776	1,267	0.16
**Total (km^2)**	957,113	8,911	8,100	

Table S3.

Max and median slope (in degrees) of anthropogenically connected and disconnected floodplain areas in the Lower Mississippi basin (HUC2 no. 8).

	**Anthropogenically connected**	**Anthropogenically disconnected**
Max slope per area (mean)	2.3	2.3
Max slope per area (median)	1.02	1.18
Median slope per area (mean)	1.25	1.58
Median slope per area (median)	0.47	0.84

Table S4.
Percent of land use using the 2016 NLCD in anthropogenically connected and disconnected, and agreement floodplains for the CONUS.

Land use	Anthropogenically connected	Anthropogenically disconnected	Agreement
Barren land	0	0	3
Cultivated crops	47	36	18
Hay pasture	8	11	6
Deciduous forest	7	7	4
Evergreen forest	2	6	4
Mixed forest	2	3	1
Developed high intensity	1	1	0
Developed low intensity	3	4	2
Developed medium intensity	2	2	1
Developed open space	5	5	3
Emergent herbaceous wetlands	3	3	7
Woody wetlands	12	8	17
Herbaceous	4	7	7
Open water	2	2	17
Perennial snow ice	0	0	0
Shrub and scrub	2	5	10
Unclassified	0	0	0

REFERENCES AND NOTES

1. T. Dunne, R.E. Aalto, Large river floodplains, in *Treatise on Geomorphology,* J. Shroder (Editor in Chief), E. Wohl (volume editor), editors. (Academic Press, 2013), vol. 9, pp. 645–678.

2. J. Harvey, M. Gooseff, River corridor science: Hydrologic exchange and ecological consequences from bedforms to basins. *Water Resour. Res.* **51**, 9, 6893–6922 (2015).

3. S.L. Dingman, R.H. Platt, Floodplain zoning: Implications of hydrologic and legal uncertainty. *Water Resour. Res.* **14**, 519–523 (1977).

4. C.J. Woltemade, K.W. Potter, A watershed modeling analysis of fluvial geomorphologic influences on flood peak attenuation. *Water Resour. Res.* **30**, 1933–1942 (1994).

5. L.A. Mertes, Documentation and significance of the perirheic zone on inundated floodplains. *Water Resour. Res.* **33**, 1749–1762 (1997).

6. W. J. Junk, P. B. Bayley, R. E. Sparks, The flood pulse concept in river–floodplain systems. *Can. Spec. Publ. Fish. Aquat. Sci.* **106**, 110–127 (1989).

7. M. G. Wolman, L. B. Leopold, "River flood plains: Some observations on their formation" (USGS Professional Paper 282-C, Government Printing Office, 1957), pp. 87–107.

8. M. G. Wolman, J. P. Miller, Magnitude and frequency of forces in geomorphic processes. *J. Geol.* **68**, 54–74 (1960).

9. G. C. Nanson, J. C. Croke, A genetic classification of floodplains. *Geomorphology* **4**, 459–486 (1992).

10. T. Dunne, L. A. Mertes, R. H. Meade, J. E. Richey, B. R. Forsberg, Exchanges of sediment between the flood plain and the channel of the Amazon River in Brazil. *Geol. Soc. Am. Bull.* **110**, 450–467 (1998).

11. G. F. White, Water science and technology: Some lessons from the 20th century. *Environ. Sci. Policy Sustain. Develop.* **42,** 30–38 (2000).

12. R. E. Sparks, Need for ecosystem management of large rivers and their floodplains. *Bioscience* **45**, 168–182 (1995).

13. E. E. Wohl, B. P. Bledsoe, R. B. Jacobson, N. L. Poff, S. L. Rathburn, D. M. Walters, A. C. Wilcox, The natural sediment regime in rivers: Broadening the foundation for ecosystem management. *Bioscience* **65**, 358–371 (2015).

14. F. Nardi, R. R. Morrison, A. Annis, T. E. Grantham, Hydrologic scaling for hydrogeomorphic floodplain mapping: Insights into human-induced floodplain disconnectivity. *River Res. Appl.* **34**, 675–685 (2018).

15. A. Annis, F. Nardi, R. R. Morrison, F. Castelli, Investigating hydrogeomorphic floodplain mapping performance with varying DTM resolution and stream order. *Hydrol. Sci. J.* **64**, 525–538 (2019).

16. O. E. Wing, P. D. Bates, C. C. Sampson, A. M. Smith, K. A. Johnson, T. A. Erickson, Validation of a 30 m resolution flood hazard model of the conterminous United States, *Water Resour. Res.* **53**, 7968–7986 (2017).

17. F. Nardi, A. Annis, G. Di Baldassarre, E. R. Vivoni, S. Grimaldi, GFPLAIN250m, a global high-resolution dataset of Earth's floodplains. *Sci. Data* **6**, 180309 (2019).

18. K. Scheel, R. R. Morrison, A. Annis, F. Nardi, Understanding the large-scale influence of levees on floodplain connectivity using a hydrogeomorphic approach. *J. Am. Water Resour. Assoc.* **55**, 413–429 (2019).

19. A. Annis, M. Karpack, R. R. Morrison, F. Nardi, On the influence of river basin morphology and climate on hydrogeomorphic floodplain delineations. *Adv. Water Resour.* **159**, 104078 (2022).

20. F. Nardi, E. R. Vivoni, S. Grimaldi, Investigating a floodplain scaling relation using a hydrogeomorphic delineation method. *Water Resour. Res.* **42**, W09409 (2006).

21. S. Lindersson, L. Brandimarte, J. Mård, G. Di Baldassarre, Global riverine flood risk—How do hydrogeomorphic floodplain maps compare to flood hazard maps? *Nat. Hazards Earth Syst. Sci.* **21**, 2921–2948 (2021).

22. P. F. Hudson, H. Middelkoop, E. Stouthamer, Flood management along the Lower Mississippi and Rhine Rivers (The Netherlands) and the continuum of geomorphic adjustment. *Geomorphology* **101**, 209–236 (2008).

23. G. Sofia, G. D. Fontana, P. Tarolli, High-resolution topography and anthropogenic feature extraction: Testing geomorphometric parameters in floodplains. *Hydrol. Process.* **28**, 2046–2061 (2014).

24. P. Blanton, W.A. Marcus, Railroads, roads and lateral disconnection in the river landscapes of the continental United States. *Geomorphology* **112**, 212–227 (2009).

25. E. E. Wohl, Human alterations of rivers, in *Sustaining River Ecosystems and Water Resources* (Springer, 2018), pp. 59–104.

26. W. L. Graf, Dam nation: A geographic census of American dams and their large-scale hydrologic impacts. *Water Resour. Res.* **35**, 1305–1311 (1999).

27. W. L. Graf, Damage control: Restoring the physical integrity of America's rivers. *Ann. Am. Assoc. Geogr.* **91**, 1–27 (2001).

28. C. Nilsson, C. A. Reidy, M. Dynesius, C. Revenga, Fragmentation and flow regulation of the world's large river systems. *Science* **308**, 405–408 (2005).

29. N. L. Poff, J. D. Allen, M. B. Bain, J. R. Karr, K. L. Prestegaard, B. D. Richter, R. E. Sparks, J. C. Stromberg, The natural flow regime. *Bioscience* **47**, 769–784 (1997).

30. C. J. Vörösmarty, M. Meybeck, B. Fekete, K. Sharma, P. Green, J. P. Syvitski, Anthropogenic sediment retention: Major global impact from registered river impoundments. *Glob. Planet. Change* **39**, 169–190 (2003).

31. J. P. Syvitski, C. J. Vörösmarty, A. J. Kettner, P. Green, Impact of humans on the flux of terrestrial sediment to the global coastal ocean. *Science* **308**, 376–380 (2005).

32. J. S. Hecht, G. Lacombe, M. E. Arias, T. D. Dan, T. Piman, Hydropower dams of the Mekong River basin: A review of their hydrological impacts. *J. Hydrol.* **568**, 285–300 (2019).

33. J. C. Stout, P. Belmont, TerEx Toolbox for semi-automated selection of fluvial terrace and floodplain features from lidar. *Earth Surf. Process. Landf.* **39**, 569–580 (2014).

34. K. Jafarzadegan, V. Merwade, A DEM-based approach for large-scale floodplain mapping in ungauged watersheds. *J. Hydrol.* **550**, 650–662 (2017).

35. R. L. Knox, R. R. Morrison, E. E. Wohl, Identification of artificial levees in the Contiguous United States. *Water Resour. Res.* **58**, e2021WR031308 (2022).

36. A. N. Strahler, Quantitative analysis of watershed geomorphology. *Eos* **38**, 913–920 (1957).

37. G. A. Tobin, The levee love affair: A stormy relationship? *J. Am. Water Resour. Assoc.* **31**, 359–367 (1995).

38. R. E. Criss, E. L. Shock, Flood enhancement through flood control. *Geology* **29**, 875–878 (2001).

39. R. A. Heine, N. Pinter, Levee effects upon flood levels: An empirical assessment. *Hydrol. Process.* **26**, 3225–3240 (2012).

40. W. Czech, A. Radecki-Pawlik, B. Wyżga, H. Hajdukiewicz, Modelling the flooding capacity of a Polish Carpathian river: A comparison of constrained and free channel conditions. *Geomorphology* **272**, 32–42 (2016).

41. A. Rajib, Q. Zheng, H. E. Golden, Q. Wu, C. R. Lane, J. R. Christensen, R. R. Morrison, A. Annis, F. Nardi, The changing face of floodplains in the Mississippi River Basin detected by a 60-year land use change dataset. *Sci. Data* **8**, 271 (2021).

42. Esri Inc., ArcGIS Pro (Version 2.8.3) (2021); www.esri.com/en-us/arcgis/products/arcgis-pro/overview.

43. R Core Team, R: A language and environment for statistical computing (R Foundation for Statistical Computing, 2020); www.R-project.org/.

44. L. Henry, H. Wickham, purrr: Functional programming tools. R package version 0.3.4 (2020); https://CRAN.R-project.org/package=purrr.

45. H. Wickham, R. François, L. Henry, K. Müller, dplyr: A Grammar of Data Manipulation. R package version 1.0.2 (2020); https://CRAN.R-project.org/package=dplyr.

46. R. L. Knox, R. R. Morrison, E. E. Wohl, CONUS floodplain areas (HydroShare, 2022); https://doi.org/10.4211/hs.15c4ab0ebfe7447298b18af37caf4e0e.

47. F. Nardi, C. Biscarini, S. Di Francesco, P. Manciola, L. Ubertini, Comparing a large-scale DEM-based floodplain delineation algorithm with standard flood maps: The Tiber River Basin case study. *Irrig. Drain.* **62**, 11–19 (2013).

48. L. B. Leopold, T. Maddock, "The hydraulic geometry of stream channels and some physiographic implications" (USGS Professional Paper 252, Government Printing Office, 1953).

49. D. Gesch, M. Oimoen, S. Greenlee, C. Nelson, M. Steuck, D. Tyler, The national elevation dataset. *Photogramm. Eng. Remote Sens.* **68**, 5–32 (2002).

50. S. G. Buto, R. D. Anderson, "NHDPlus High Resolution (NHDPlus HR)—A hydrography framework for the Nation" (no. 2020-3033, U.S. Geological Survey, 2020).

51. P. R. Seaber, F. P. Kapinos, G. L. Knapp, "Hydrologic unit maps" (USGS Water Supply Paper 2294, U.S. Geological Survey, 1987).

52. S. Jin, C. Homer, L. Yang, P. Danielson, J. Dewitz, J. L. Congcong, Z. Zhu, G. Xian, D. Howard, Overall methodology design for the United States national land cover database 2016 products. *Remote Sens. (Basel)* **11**, 2971 (2019).
